# Meta-Analysis Suggests Differing Indirect Effects of Viral, Bacterial, and Fungal Plant Pathogens on the Natural Enemies of Insect Herbivores

**DOI:** 10.3390/insects11110765

**Published:** 2020-11-06

**Authors:** Ussawit Srisakrapikoop, Tara J. Pirie, Mark D. E. Fellowes

**Affiliations:** People and Wildlife Research Group, School of Biological Sciences, University of Reading, Whiteknights, Reading, Berkshire RG6 6AJ, UK; u.srisakrapikoop@pgr.reading.ac.uk (U.S.); t.pirie@reading.ac.uk (T.J.P.)

**Keywords:** tritrophic systems, plant-mediated indirect interactions, consumptive effects, non-consumptive effects, parasitoid, predator

## Abstract

**Simple Summary:**

Plants suffer from disease, caused by fungal, bacterial and viral pathogens. Some insect herbivores avoid diseased plants, while others are attracted to them, often depending on how infection changes the quality of the plant. In turn, the herbivores are attacked by enemies, such as insect predators and parasitoids. Few studies have investigated how plant pathogens affect the herbivores’ insect natural enemies. Using a statistical technique called meta-analysis, we examined 216 measured responses from 29 studies, to see if we could find any patterns. The effect on natural enemies depended on the cause of the disease and type of natural enemy. Fungal pathogens had negative effects on the preference and performance of insect natural enemies, mainly through the disease reducing the quality of their insect herbivore prey. Bacterial pathogens had a positive effect on insect natural enemies, possibly caused by changes in host plant traits. We found no clear effect with viral diseases. We show that the causes of plant disease may have very different effects on ecological interactions between insect herbivores and their enemies, particularly parasitic wasps. This will help us understand how disease can change the patterns of interactions between species in natural and agricultural ecosystems.

**Abstract:**

Indirect effects are ubiquitous in nature, and have received much attention in terrestrial plant–insect herbivore–enemy systems. In such tritrophic systems, changes in plant quality can have consequential effects on the behavior and abundance of insect predators and parasitoids. Plant quality as perceived by insect herbivores may vary for a range of reasons, including because of infection by plant pathogens. However, plant diseases vary in their origin (viral, bacterial or fungal) and as a result may have differing effects on plant physiology. To investigate if the main groups of plant pathogens differ in their indirect effects on higher trophic levels, we performed a meta-analysis using 216 measured responses from 29 primary studies. There was no overall effect of plant pathogens on natural enemy traits as differences between pathogen types masked their effects. Infection by fungal plant pathogens showed indirect negative effects on the performance and preference of natural enemies via both chewing and piercing-sucking insect herbivore feeding guilds. Infection by bacterial plant pathogens had a positive effect on the natural enemies (parasitoids) of chewing herbivores. Infection by viral plant pathogens showed no clear effect, although parasitoid preference may be positively affected by their presence. It is important to note that given the limited volume of studies to date on such systems, this work should be considered exploratory. Plant pathogens are very common in nature, and tritrophic systems provide an elegant means to examine the consequences of indirect interactions in ecology. We suggest that further studies examining how plant pathogens affect higher trophic levels would be of considerable value.

## 1. Introduction

Plant pathogens are exceptionally common in terrestrial ecosystems, and have considerable economic impact in agroecosystems. Despite this, they have received surprisingly little attention from ecologists studying their wider ecological effects. For example, plant pathogens may alter host plant quality as perceived by insect herbivores, but what are the indirect consequences for species at higher trophic levels, such as the herbivores’ insect predators and parasitoids?

Studies show that plant pathogens can alter host plant quality as perceived by insect herbivores by altering nutritional value [1], volatiles [2], defensive compounds [3] and appearance [4], and these affect herbivore life histories via both individual performance and preference [5,6,7]. Changes in plant quality also affects the dynamics of insect herbivores and their natural enemies [8,9], and pathogen infection can cause similar changes by also altering plant quality. This consequence of infection can be categorized as an indirect effect as it is the result of the interaction between two species, mediated by at least one intermediate species in the chain [10]. However, the results of studies of the indirect effects of plant pathogens on insect natural enemies are inconsistent. Some studies found positive [5,11] effects of infection on natural enemy preference and performance, while others show negative consequences of plant pathogen infection [7,12]. An general understanding of how plant pathogen infection may influence the indirect interactions between plants and the natural enemies of their insect herbivores remains largely undeveloped, a knowledge gap that this paper begins to address.

One key link between plants and the natural enemies of their herbivores comes from the effects of plant defenses on their insect herbivores. Plants have to defend themselves against attack, and there are two primary hormonal signaling pathways that facilitate defense: the salicylic acid (SA) and the jasmonic acid (JA) pathways [13]. The SA signaling pathway is activated against piercing-sucking herbivores, biotrophic pathogens and viruses, while the JA signaling pathway is activated against chewing herbivores, necrotrophic pathogens and bacteria [14]. As a result, infection by a pathogen, or attack by a herbivore, may result in differing defenses being elicited, defenses which affect more than one group of plant natural enemies.

Such changes in plant quality may have indirect consequences for the herbivores’ insect natural enemies. The preference–performance hypothesis contends that insect herbivore performance matches their host plant preferences [15], a suggestion which has received support [16,17], albeit not always [18,19]. Few studies have considered similar effects at higher trophic levels, where there is again some support for this idea [7,20], but this is not always so [21,22,23,24].

Here we consider the indirect effects of plant pathogens on insect natural enemies. Plant pathogens may modify the phenotypic responses of infected plants and in affect their herbivores, which then in turn influence herbivore–enemy interactions. These effects can be classified into consumptive and non-consumptive pathways; with the former the effects are transmitted through the consumption of herbivores, while with the latter the effects are transmitted through changes in appearance and/or volatiles from plants and herbivores as perceived by natural enemies.

Furthermore, the natural enemies themselves may differ in their responses due to differences in life histories. For example, parasitoid development is intimately associated with individual hosts, and so the quality of a single host may have a substantial effect on an individual parasitoid’s fitness. In contrast, insect predators may simply be able to compensate for poor quality hosts by consuming more prey.

The objectives of this study were (1) to investigate the overall effect of plant pathogen infection on natural enemies; and (2) to examine if pathogen type, herbivore feeding guild, natural enemy guild (predator or parasitoid), natural enemy responses (performance and preference) and their interactions affect natural enemies. To answer these questions, we systematically reviewed the literature and used metanalytical techniques.

## 2. Materials and Methods

### 2.1. Data Collection

We searched for published articles reporting the indirect effects of plant pathogen infection on insect natural enemies in the Web of Science database (ISI) by applying the following search terms “(Plant or tree) and (predator* or *parasitoid* or natural enem*) and (preference or performance or choice or indirect effect*) and (fung* or oomyc* or bacteri* or virus or plant pathogen* or plant infection) not *mycorrh* not endophyt* not *symbio* not entomopathogen*”, where * refers to a ‘wild card’ entry. The search was refined by showing only the studies published in articles, proceedings papers, reviews and book chapters. The search yielded 423 papers and the other 25 papers were found from references in the reviews, book chapters and in press articles (see the PRISMA flow diagram, Supplementary Materials S1).

The studies included in the meta-analysis had to meet the following criteria: they (1) report natural enemy performance or preference toward either healthy versus pathogen-infected plants or insect herbivores feeding on healthy versus pathogen-infected plants; (2) report plant, insect herbivore, pathogen at species level and natural enemy to at least family level and (3) report the mean and variability (standard deviation, standard error or confidence interval) with sample size. When necessary, ImageJ was used to extract mean and variability from figures. This yielded 216 case studies from 29 studies (note that three studies came from a PhD thesis [25]). The references of the primary studies included in this study are shown in Supplementary Materials S2.

### 2.2. Moderators

For each case study, the following moderators were extracted according to the pathogen, insect herbivore, type and process of insect natural enemy response; pathogen types (bacterial, fungal or viral); insect herbivore feeding guild (chewing or piercing-sucking); experimental conditions (laboratory or field); type of natural enemy (predator or parasitoid); type of natural enemy response (performance or preference); mechanisms of the indirect effects originating from plant pathogens that are relayed to natural enemies (consumptive via herbivore (CH), non-consumptive via herbivore (NH), non-consumptive via plant (NP) and non-consumptive via herbivore and plant (NHP)).

**Figure 1:**
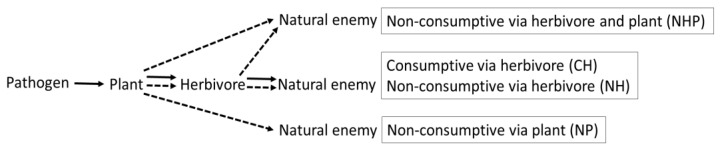
The dissemination processes of indirect effects originating from plant pathogens relaying to natural enemies. A filled arrow represents a direct effect and a dashed arrow represents an indirect effect.

The definition of preference in this study is any trait exhibited by natural enemies where more than one stimulus is given at the same time and the insects make a choice, whereas performance is any trait exhibited by natural enemies where no choice is given. For mechanisms, the indirect effect can (a) originate from plant pathogens relayed to natural enemies mediated by the consumption of insect herbivore prey (CH), or (b) through indirect effects mediated by changes in appearance and/or volatiles emanating from the herbivore (NH), the host plant (NP) or both herbivore and plant together (NHP). These mechanisms were categorized based on the dissemination processes (Figure 1) of the effects and experimental design of primary studies. The cases where natural enemy performance was measured after the consumption of insect herbivore prey (e.g., longevity, development time, fecundity, weight, size) were categorized as CH, whereas in some cases the natural enemy performance (no choice given) or preference (choice given) were measured through non-consumptive traits (e.g., proportion of parasitized hosts, time spent around herbivores, number of antennation events, time to first attack) were categorized as NH. Studies conducted only with the host plant were classified as NP, while the cases where insect herbivores are present on host plants were classified as NHP. We differentiated NHP from NP as in some measured responses, both herbivore and host plant were present, so it is uncertain that the indirect effects affecting the natural enemies come only from changes in the herbivore, the plant or both. All of these processes can help elucidate the mechanism by which the indirect effects from plant pathogens can be relayed to natural enemies.

To avoid the confounding effect of the non-independence of data resulting from bias due to the nested experimental design in the same studies, each published article was given a *Study ID*, *Case ID* and *System ID* [26]. Each published article was given an identifier (*Study ID*), in which each study can contain more than one measured response. Each measured response was a response variable of a natural enemy towards a paired treatment (healthy versus infected plants, or insect herbivores feeding on healthy versus infected plants), in which each measured response was given a unique ID (*Case ID*). In addition, *System ID* was given to the combination of plant pathogen, plant, insect herbivore and insect natural enemy.

### 2.3. Statistical Analyses

All statistical analyses were performed in R 4.0.2 [27] using the *metafor* package [28]. The effect size of each measured response was calculated using Hedges’ *d* metric and its variance [29] (for effect size calculations refer to Supplementary Materials S3). All models were performed using multilevel linear mixed-effect models (with Restricted Maximum Likelihood (REML)) to avoid confounding effects from non-dependent data as most studies contained more than one measured response, and these measured responses from a single study tended to be correlated. As measured responses from the same study system were likely to be correlated, *System ID* was used as a random factor as well as *Case ID* nested in *Study ID* was used as another random factor [26].

Grand mean effect size was calculated using the complete data set. Then subgroup analyses were performed. First, the effect of different experiment conditions (laboratory versus field) was tested, and found no influence of different experiment conditions on effect size. Therefore, data from laboratory and field experiments were pooled for the following subgroup analyses. Note that in subgroup analyses some levels of moderators were combined with other levels of moderator or excluded from the subgroup analyses as appropriate. One study with oomycete as a plant pathogen was assigned into the fungus group as well as the feeding guild; a study with thrips as an insect herbivore was assigned into the chewing group. Two field studies, one in which the insect herbivore was a seed predator caterpillar (the indirect effect arises from change in plant phenotype by infection rather than the effect from herbivore feeding; [30]) and another with a mixed type of herbivores (a field study in which insect herbivores naturally occurred without manipulation; [4]) were excluded from the subgroup analyses where feeding guild was a moderator. Additionally, in some subgroup analyses there were unbalanced measured responses as there were no measured responses for some levels of some moderators (Supplementary Materials S4).

Publication bias was tested by (1) inspection of funnel plots, (2) conducting cumulative meta-analysis [31], (3) calculation of fail-safe number [32] and (4) detecting the relationship between effect sizes and journal impact factor [33]. For sensitivity analysis, Cook’s distances were considered and studentized deleted residual values greater than |3.0| were excluded from the analysis [34].

## 3. Results

We can identify 40 primary studies examining the indirect effects of plant pathogen infection on insect natural enemies, but only 29 primary studies that reported mean and variability were suitable for inclusion in the study. These consisted of 18 plant species, 15 insect herbivore species, 22 plant pathogen species (bacteria (3), fungus (10) and virus (9)) and 19 natural enemy species (parasitoid (11), predatory mite (2), predatory bug (2), lacewing (2) and ladybird (2)).

The grand mean effect size (±95% Confidence Intervals) after outliers were removed (*k* = 213) was not significant (*p* = 0.187) with −0.21 ± CI (−0.51, 0.10). This indicated that there was no indirect effect of plant pathogens on insect natural enemies. However, the significance value and large amount of residual heterogeneity indicated that other moderators which were not included in the initial model may influence effects on natural enemies (*Q*_E_ = 2213.36, *df* = 212, *p* < 0.0001). The estimated means for laboratory (*k* = 183, −0.12 ± CI (−0.47, 0.22)) and field (*k* = 29, −0.3924 ± CI (−1.02, 0.24)) studies were not significantly different from each other (*p* = 0.46) and experimental condition was not a significant moderator (*Q*_M_ = 1.96, *df* = 2, *p* = 0.38), so data from laboratory and field studies were pooled.

**Figure 2:**
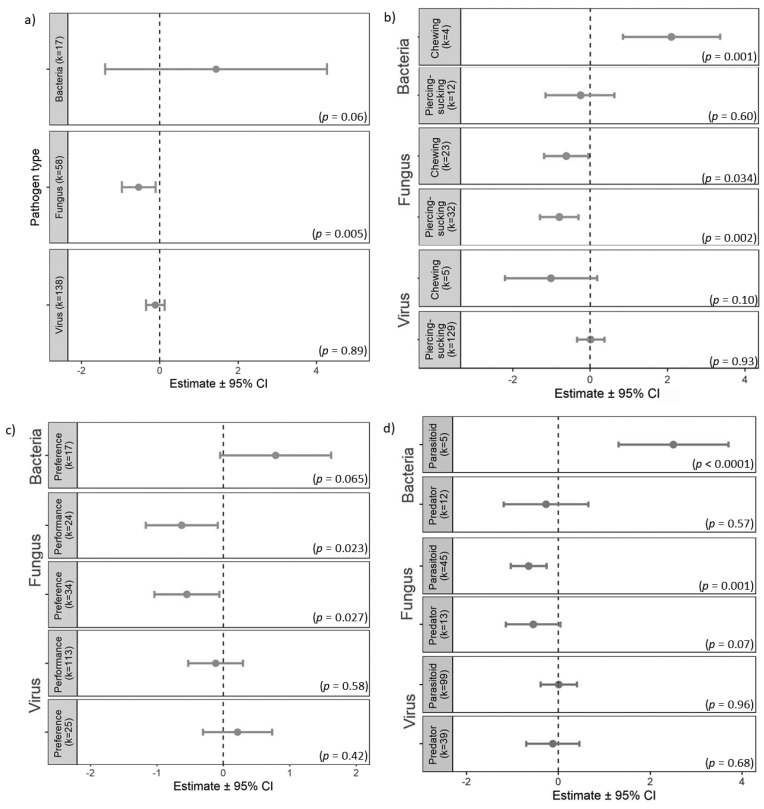
Natural enemy responses toward the indirect effects from plant pathogen infection by (**a**) pathogen type (virus, fungus, or bacteria); (**b**) interaction of insect herbivore feeding guild (piercing-sucking or chewing) and pathogen type; (**c**) interaction of type of natural enemy response (preference or perfomance) and pathogen type and (**d**) interaction of pathogen type and natural enemy type (predator or parasitoid). Circles and error bars represent the estimates and corresponding 95% CI. The vertical dashed line at zero represents the null hypothesis (no difference in natural enemy response between control and infected treatments).

The indirect effects of plant pathogens on natural enemies was significantly different between pathogen types (*Q*_M_ = 11.43, *df* = 3, *p* = 0.0096). Only fungal pathogens caused significant negative indirect effects on natural enemies, while the effects from bacteria and viruses were not significant (Figure 2a). However, there was a significant effect of the interaction between insect herbivore feeding guild and pathogen type (pathogen × feeding guild: *Q*_M_ = 28.13, *df* = 6, *p* < 0.0001). The interaction between bacterial pathogen infection and chewing insect guild caused a significant positive indirect effect on natural enemies, while the interactions for both chewing and piercing-sucking insect guilds with fungal pathogens were negatively significant (Figure 2b). There was also a significant interaction between type of responses and pathogen type (*Q*_M_ = 12.40, *df* = 5, *p* = 0.03), in that the fungal pathogen caused significant indirect negative effects on natural enemy performance and preference (Figure 2c).

When considering the overall indirect effects of plant pathogens on natural enemy types, natural enemy type as a moderator was not significant (*Q*_M_ = 1.72, *df* = 2, *p* = 0.42; estimated mean for predator: *k* = 65, −0.22 ± (−0.67, 0.23); parasitoid *k* = 148, −0.20 ± (−0.54, 0.14)). However, we found a significant interaction between pathogen type and natural enemy type (*Q*_M_ = 28.68, *df* = 6, *p* < 0.0001) where a positive indirect effect was found in the Parasitoid × Bacteria interaction (2.51 ± (1.31, 3.70), *p* < 0.0001), and a negative indirect effect was found in the Parasitoid × Fungus interaction (−0.65 ± (−1.04, −0.26), *p* = 0.0011) (Figure 2d).

We also examined the interaction effect between pathogen types and mechanisms (pathogen × mechanism) on how the indirect effects from plant pathogens are passed to natural enemies. This interaction was significant (*Q*_M_ = 30.88, *df* = 10, *p* = 0.0006) where the negative effects from fungal pathogens via CH and NHP were significant (Fungus × CH: −0.72 ± (−1.33, −0.11), *p* = 0.021; Fungus × NHP: −0.53 ± (−1.04, −0.02), *p* = 0.04). The positive indirect effect from viruses via NP was also significant (Virus × NP: 7.66 ± (2.60, 12.72), *p* = 0.003).

Finally, the pathways by which the indirect effects from plant pathogens are relayed, and their effect on natural enemies, were examined through the three-way interaction between pathogen type, mechanism and response type (pathogen × mechanism × response). This three-way interaction was significant (*Q*_M_ = 29.53, *df* = 14, *p* = 0.0089) and the significant terms were similar to the previous model (pathogen × mechanism). The significant terms were Fungus × CH × Performance (−0.71 ± (−1.35, −0.06), *p* = 0.032) and Virus × NP × Preference (7.58 ± (2.49, 12.66), *p* = 0.0035).

Publication bias was detected in a funnel plot with visual assessment (Supplementary Materials S5), but the Rosenberg’s fail-safe number of 11,095 exceeded the critical conservative value of 5 × k +10 = 1100, suggesting that our results are robust [32]. There was evidence of temporal change in cumulative meta-analysis (Supplementary Materials S6). The early studies of indirect effects from plant pathogens on natural enemies revealed the effects tended to be positive with large variance. However, results became negative over time with smaller variance. We found no correlation between effect sizes and journal impact factor (Spearman’s rho = −0.0043, *p* = 0.95).

## 4. Discussion

Forty primary studies that investigated the indirect effects of plant pathogens on natural enemies were identified, of which the first was published in 1998 [35]. This suggests that during the last 22 years there have been relatively few studies that have considered the indirect effects of plant pathogen infection on the natural enemies of insect herbivores. Given that (a) plant pathogens are widespread, ecologically and economically important, and affect their quality as host plants for herbivores, and (b) that indirect effects, such as those found in tritrophic systems, are ecologically important and ubiquitous, then we suggest that the intersection of these two observations is likely to be of considerable interest to pure and applied ecologists. Overall, we found no significant effect of pathogen infection on the insect natural enemies of plant herbivores, but when we consider the pathogen types separately, we find that only fungal plant pathogens cause indirect negative effects on natural enemies. Viral pathogens show no consistent effect, but bacterial pathogens appear to benefit the enemies of chewing herbivores. Breaking this down further to consider parasitoids and insect predators, we found no clear indirect effects of plant pathogens on predators, but host plant infection by fungal plant pathogens has a negative effect and infection by bacterial pathogens has a positive effect on parasitoids. It should be noted that any meta-analysis is only as good as the data used in the synthesis. We find some evidence of publication bias, which given the number of available studies is perhaps unsurprising. We therefore conservatively see these results as indicative of the effects of plant pathogens on interactions at higher trophic levels, but further work is required to build on this evidence base.

The negative influence of fungal plant pathogens on insect natural enemies is consistent with a previous study [26]. We found that both the preference and performance of insect natural enemies was affected by fungal pathogen infection, and that natural enemies of both piercing-sucking and chewing herbivores were negatively affected. Insect herbivores feeding on fungus-infected plants are likely to suffer from changes in plant nutritional value [1,36], plant defensive compounds from pathogen infection (induced resistance) [3] and possibly mycotoxins resulting from fungal infection [37]. All of these lead to poorer performance of the herbivores, which in turn is relayed to natural enemies due to poorer prey/host quality.

In contrast to the effects of fungal pathogens, bacterial pathogens can cause significant positive indirect effects on natural enemies via chewing insect prey. We note however, that this result comes from four measured responses, all resulting from one primary study [38]. The four measured responses were preference studies with plant (NP) and herbivore and plant (NHP) mediated effects. The study found similar plant volatile responses between herbivory and infection, and infection alone was enough to induce a change in the natural enemy’s behavior [38]. Further studies are needed to confirm the generality of this finding. In addition, we suggest that further studies examining the effect of bacterial plant pathogens on natural enemy preference (where the non-significant trend was to a positive effect) and performance (where we lack studies) would be of considerable value. Finally, we found no consistent effect of viral plant pathogen infection on the preference and performance of insect natural enemies. Unlike the situation with bacterial plant pathogens, this was not due to a lack of studies.

Indeed, if we consider parasitoids and insect predators separately, a pattern emerges. While again acknowledging that there are few studies, we found no significant indirect effect of plant pathogens on insect predators. In contrast, and given that parasitoid fitness is tightly associated with host quality (and indeed host plant quality, e.g., [2,7,25]), we found an effect of plant pathogen infection on parasitoids. However, while fungal plant pathogens had a negative effect, bacterial plant pathogens (although this evidence comes from just two primary studies [5,38]) had a positive effect, attracting parasitoids to infected plants. This is an area which would greatly benefit from further study. Additionally, while we find an overall effect of fungal plant pathogen infection on natural enemies, we suggest that different fungal pathogen life styles (endophyte, biotrophic and necrotrophic pathogens) may contribute to different responses of insect herbivores [26]. Fungal infection can harm insect herbivores by both direct effect from mycotoxin produced [37] and indirect effects mediated by host plants from changing plant nutrition [36], relocation and sink source of nutrients, and changing in plant defense responses [39]. All of these lead to detrimental effects on herbivore performances such as smaller herbivore size leading to smaller natural enemy size [7]. Likewise, our result indicated that fungal infection changes natural enemy preference by repelling them from infected plants.

Generally, natural enemies often employ chemical cues associated with insect herbivory such as herbivore-induced plant volatiles (HIPVs) to locate potential prey/host [40]. Very few studies show that natural enemies substantially prefer herbivore-damaged plants over fungus-infected plants. However, when herbivore-damaged plants were compared with plants with both herbivory and fungal infection, then these two treatments were not statistically different [2,21]. Fungal infection can reduce the production of green leaf volatiles (GLVs), glucosinolate derivatives and terpenoids after herbivory [2], and these volatiles are important in attracting natural enemies [41].

We hypothesized that different pathogen types may have different pathways to relay the indirect effects of disease to insect natural enemies. We found that fungal infection contributes indirect negative effects to natural enemies via CH and NHP. The effect of fungal pathogens on natural enemies via the CH mechanism is straightforward, as feeding on poor quality hosts contributes to detrimental effects on the natural enemy. One study found that aphids feeding on fungus-infected plants were less resistant to starvation and the parasitoids emerged from the aphids feeding on the infected plants also showed reduced starvation resistance [7]. The evidence of fungal infection affecting natural enemies via NPH may be explained by changes in plant volatiles.

Viral plant pathogen infection significantly caused an indirect positive effect on natural enemies (parasitoid) via NP (albeit from one measured response [6]) and this means virus-infected plants attract natural enemies. We speculate that this attraction may arise from changes in plant volatiles even though we have no supporting evidence as the experimental design of the primary study did not include plant volatile analysis [6]. If we were to speculate, we would suggest that given that viral pathogens frequently rely on insect vectors for transmission, it would not be surprising if changes in volatiles which attract insect herbivores, and hence their enemies, were selected for [42]. The presence of virus-infected plants is likely to be a reliable cue to indicate the existence of potential hosts to natural enemies [43]. It is not only volatiles that are changed in virus-infected plants, but also their color [4].

## 5. Conclusions

Although plant pathogen, plant, insect herbivore and natural enemy interactions are ubiquitous in both natural and agricultural ecosystems, there has been surprisingly few studies addressing how plant pathogen infection may indirectly affect the preference and performance of insect natural enemies. Our meta-analysis suggests that the indirect effects of plant pathogens on insect natural enemies varies with both pathogen type, natural enemy type, and through the route by which the effect is transmitted. The indirect effects of infection by fungal plant pathogens were negative, and the limited number of studies using bacterial plant pathogens suggested a positive effect on parasitoid preference, although two primary studies showed a positive preference effect. Host plant infection by viral pathogens did not show such effects, although a transmission pathway was detected. It is therefore not simply that being a diseased plant alters the preference and performance of associated parasitoids and insect predators. Instead, it is an interaction between the causative agent of disease, combined with herbivore and enemy traits, which determines the ecological outcome. We hope that this meta-analysis highlights the knowledge gap that exists in our understanding of the ecological consequences of plant disease. Further work will help us understand if the conclusions that can be drawn from the limited number of available studies are robust. We suggest that studies in this neglected area will be of considerable interest to both ecologists and pest managers.

## Supplementary Materials

The following are available online at https://www.mdpi.com/2075-4450/11/11/765/s1, Supplementary Materials S1: PRISMA flow chart, Supplementary Materials S2: List of primary studies included in this quantitative synthesis, Supplementary Materials S3: Effect size calculation, Supplementary Materials S4: The number of case studies following given moderators, Supplementary Materials S5: Funnel plot, Supplementary Materials S6: Cumulative meta-analysis.
