# Supplementary material for: Meta-Analysis Suggests Differing Indirect Effects of Viral, Bacterial, and Fungal Plant Pathogens on the Natural Enemies of Insect Herbivores
Source: Insects. 2020 Nov 6;11(11):765. doi: 10.3390/insects11110765 (PMC7694682; doi:10.3390/insects11110765)
Supplement: Supplementary file 1 [file insects-11-00765-s001.pdf]

Supplementary Materials

Supplementary Materials S1.

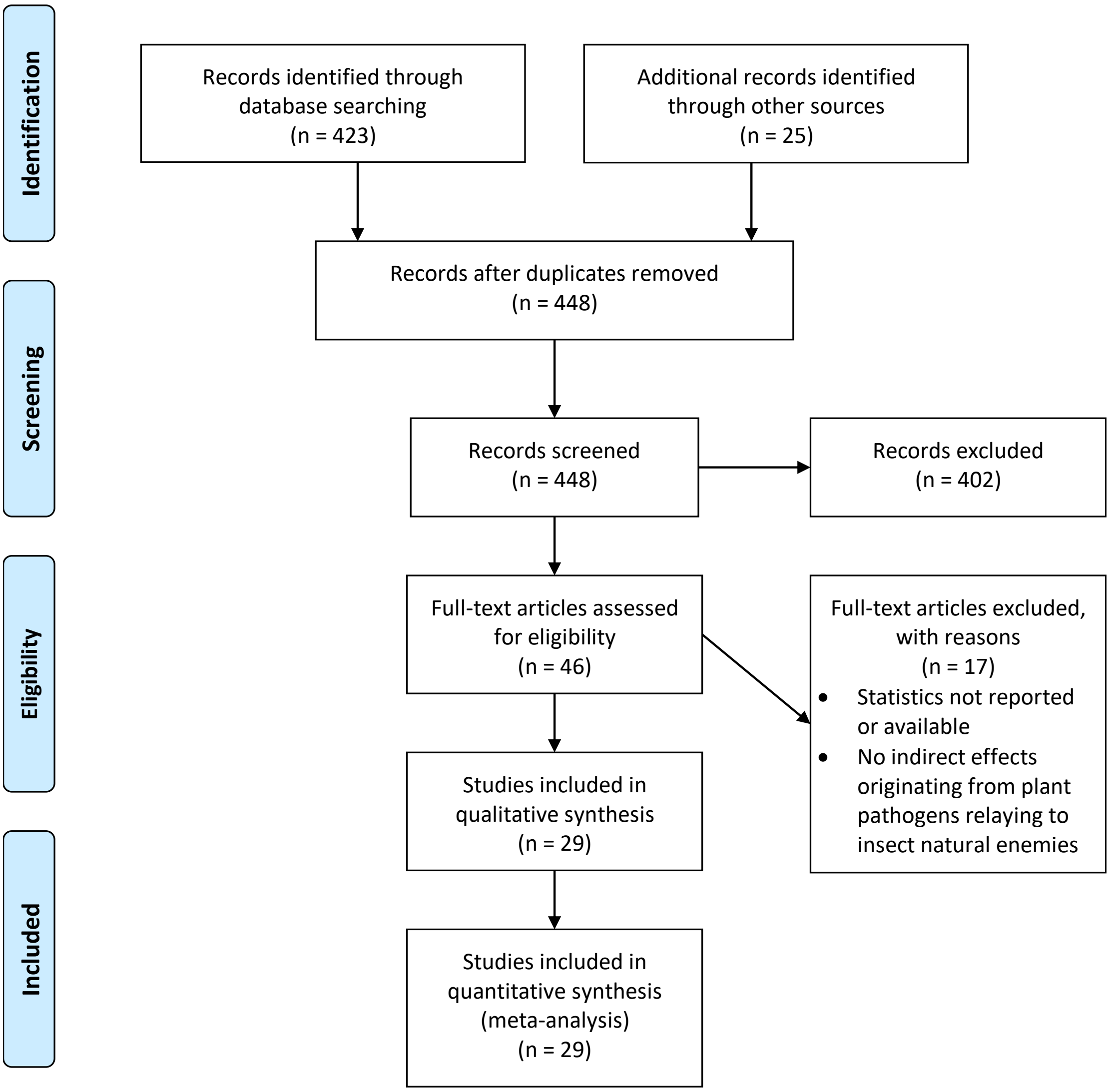

Figure S1. PRISMA Flow Chart for Our Data Set

From: Moher, D.; Liberati, A.; Tetzlaff, J.; Altman, D.G.; The PRISMA Group. Preferred Reporting Items for Systematic Reviews and Meta-Analyses: The PRISMA Statement. *PLoS Med*. **2009**, 6, e1000097, doi:10.1371/journal.pmed1000097

Supplementary Materials S2. List of Primary Studies Included in this Quantitative Synthesis

1. Calvo, D.; Fereres, A. The performance of an aphid parasitoid is negatively affected by the presence of a circulative plant virus. *BioControl* **2011**, 56, 747–757, doi:10.1007/s10526-011-9354-x.
2. de Oliveira, C.F.; Long, E.Y.; Finke, D.L. A negative effect of a pathogen on its vector? A plant pathogen increases the vulnerability of its vector to attack by natural enemies. *Oecologia* **2014**, *174*, 1169–1177, doi:10.1007/s00442-013-2854-x.
3. Sun, Z.; Liu, Z.; Zhou, W.; Jin, H.; Liu, H.; Zhou, A.; Zhang, A.; Wang, M.Q. Temporal interactions of plant - insect - predator after infection of bacterial pathogen on rice plants. *Sci. Rep.* **2016**, *6*, 1–12, doi:10.1038/srep26043.
4. de Oliveira, R.L.; Moscardini, V.F.; Gontijo, P.C.; Sâmia, R.R.; Marucci, R.C.; Budia, F.; Carvalho, G.A. Life history parameters and feeding preference of the green lacewing *Ceraeochrysa cubana* fed with virus-free and potato leafroll virus-infected *Myzus persicae*. *BioControl* **2016**, *61*, 671–679, doi:10.1007/s10526-016-9748-x.
5. Van Nouhuys, S.; Laine, A.L. Population dynamics and sex ratio of a parasitoid altered by fungal-infected diet of host butterfly. *Proc. R. Soc. B Biol. Sci.* **2008**, *275*, 787–795, doi:10.1098/rspb.2007.1588.
6. Tack, A.J.M.; Gripenberg, S.; Roslin, T. Cross-kingdom interactions matter: fungal-mediated interactions structure an insect community on oak. *Ecol. Lett.* **2012**, *15*, 177–185, doi:10.1111/j.1461-0248.2011.01724.x.
7. Cardoza, Y.J.; Teal, P.E.A.; Tumlinson, J.H. Effect of peanut plant fungal infection on oviposition preference by *Spodoptera exigua* and on host-searching behavior by *Cotesia marginiventris*. *Environ. Entomol.* **2003**, *32*, 970–976, doi:10.1603/0046-225x-32.5.970.
8. Liu, X.; He, Y.; Xie, W.; Wu, Q.; Zhang, Y.; Liu, Y.; Wang, S. Infection of tomato by tomato yellow leaf curl virus alters the foraging behavior and parasitism of the parasitoid *Encarsia formosa* on *Bemisia tabaci*. *J. Asia. Pac. Entomol.* **2018**, *21*, 548–552, doi:10.1016/j.aspen.2018.02.016.
9. Mauck, K.E.; Smyers, E.; De Moraes, C.M.; Mescher, M.C. Virus infection influences host plant interactions with non-vector herbivores and predators. *Funct. Ecol.* **2015**, *29*, 662–673, doi:10.1111/1365-2435.12371.
10. Ngah, N.; Thomas, R.L.; Shaw, M.W.; Fellowes, M.D.E. Asymptomatic host plant infection by the widespread pathogen *Botrytis cinerea* alters the life histories, behaviors, and interactions of an aphid and its natural enemies. *Insects* **2018**, *9*, 80, doi:10.3390/insects9030080.
11. Joffrey, M.; Chesnais, Q.; Spicher, F.; Verrier, E.; Ameline, A.; Couty, A. Plant virus infection influences bottom-up regulation of a plant-aphid-parasitoid system. *J. Pest Sci. (2004).* **2018**, *91*, 361–372, doi:10.1007/s10340-017-0911-7.
12. Desurmont, G.A.; Xu, H.; Turlings, T.C.J. Powdery mildew suppresses herbivore-induced plant volatiles and interferes with parasitoid attraction in *Brassica rapa*. *Plant Cell Environ.* **2016**, *39*, 1920–1927, doi:10.1111/pce.12752.
13. Ponzio, C.; Weldegergis, B.T.; Dicke, M.; Gols, R. Compatible and incompatible pathogen–plant interactions differentially affect plant volatile emissions and the attraction of parasitoid wasps. *Funct. Ecol.* **2016**, *30*, 1779–1789, doi:10.1111/1365-2435.12689.
14. Xu, H.; He, X.; Zheng, X.; Yang, Y.; Tian, J.; Lu, Z. Infection of rice plants by rice black streaked dwarf virus improves an egg parasitoid, *Anagrus nilaparvatae* (Hymenoptera: Mymaridae), of rice planthoppers. *Environ. Entomol.* **2014**, *43*, 1235–1239, doi:10.1603/en14044.
15. Garzón, A.; Freire, B.C.; Carvalho, G.A.; Oliveira, R.L.; Medina, P.; Budia, F. Development and reproduction of *Chrysoperla externa* (Hagen) (Neuroptera: Chrysopidae) fed on *Myzus persicae* (Sulzer) (Hemiptera: Aphididae) vectoring potato leafroll virus (PLRV). *Neotrop. Entomol.* **2015**, *44*, 604–609, doi:10.1007/s13744-015-0329-y.
16. Steiner, S.; Erdmann, D.; Steidle, J.L.M.; Ruther, J. Host habitat assessment by a parasitoid using fungal volatiles. *Front. Zool.* **2007**, *4*, 1–10, doi:10.1186/1742-9994-4-3.
17. He, X.; Xu, H.; Gao, G.; Zhou, X.; Zheng, X.; Sun, Y.; Yang, Y.; Tian, J.; Lu, Z. Virus-mediated chemical changes in rice plants impact the relationship between non-vector planthopper *Nilaparvata lugens* Stål and its egg parasitoid *Anagrus nilaparvatae* Pang et Wang. *PLoS One* **2014**, *9*, e105373, doi:10.1371/journal.pone.0105373.
18. Rostás, M.; Ton, J.; Mauch-Mani, B.; Turlings, T.C.J. Fungal infection reduces herbivore-induced plant volatiles of maize but does not affect naïve parasitoids. *J. Chem. Ecol.* **2006**, *32*, 1897–1909, doi:10.1007/s10886-006-9147-3.

19. Lazebnik, J.; Tibboel, M.; Dicke, M.; van Loon, J.J.A. Inoculation of susceptible and resistant potato plants with the late blight pathogen *Phytophthora infestans*: effects on an aphid and its parasitoid. *Entomol. Exp. Appl.* **2017**, *163*, 305–314, doi:10.1111/eea.12582.
20. Liu, X.; Xiang, W.; Jiao, X.; Zhang, Y.; Xie, W.; Wu, Q.; Zhou, X.; Wang, S. Effects of plant virus and its insect vector on *Encarsia formosa*, a biocontrol agent of whiteflies. *Sci. Rep.* **2014**, *4*, 1–6, doi:10.1038/srep05926.
21. Ngah, N. Asymptomatic pathogen infection alters interactions at higher trophic levels, Ph.D. dissertation, University of Reading, **2018**.
22. Li, J.; Ding, T.; Chu, D. Differential effects of two plant viruses on performance and biocontrol efficiency of *Encarsia formosa* fed on *Bemisia tabaci*. *Biol. Control* **2020**, *142*, 104166, doi:10.1016/j.biocontrol.2019.104166.
23. Lin, Y.; Lin, S.; Akutse, K.S.; Hussain, M.; Wang, L. *Diaphorina citri* induces huanglongbing-infected citrus plant volatiles to repel and reduce the performance of *Propylaea japonica*. *Front. Plant Sci.* **2016**, *7*, 1969, doi:10.3389/fpls.2016.01969.
24. Hodge, S.; Powell, G. Complex interactions between a plant pathogen and insect parasitoid via the shared vector-host: consequences for host plant infection. *Oecologia* **2008**, *157*, 387–397, doi:10.1007/s00442-008-1083-1.
25. Martini, X.; Pelz-Stelinski, K.S.; Stelinski, L.L. Plant pathogen-induced volatiles attract parasitoids to increase parasitism of an insect vector. *Front. Ecol. Evol.* **2014**, *2*, 1–8, doi:10.3389/fevo.2014.00008.
26. Belliure, B.; Janssen, A.; Sabelis, M.W. Herbivore benefits from vectoring plant virus through reduction of period of vulnerability to predation. *Oecologia* **2008**, *156*, 797–806, doi:10.1007/s00442-008-1027-9.
27. Biere, A.; Elzinga, J.A.; Honders, S.C.; Harvey, J.A. A plant pathogen reduces the enemy-free space of an insect herbivore on a shared host plant. *Proc. R. Soc. B Biol. Sci.* **2002**, *269*, 2197–2204, doi:10.1098/rspb.2002.2147.

Supplementary Materials S3. Effect Size Calculation

For each case study, we calculated effect size using the Hedges' d metric and its variance [1]:

$$d = J\frac{\overline{x} - \overline{x}_{\text{control}}}{\sqrt{\sigma}}$$

where $x_{treatment}$ refers to mean natural enemy response on infected plants and $x_{control}$ to mean natural enemy response on healthy, control plants, with

$$J = 1 - \frac{3}{4(n_{\text{treatment}} - n_{\text{control}} - 2) - 1}$$

where $n_{treatment}$ and $n_{control}$ are the sample sizes for infected and control plants and with

$$\sigma_{\text{pooled}} = \frac{(n_{\text{treatment}} - 1)\sigma^2{}_{\text{treatment}} + (n_{\text{control}} - 1)\sigma^2{}_{\text{control}}}{n + n - 2}$$

where σ refers to the variance of natural enemy response.

Hedges' d was preferred to other metrics of effect size, such as the log-response ratio, because it is corrected for bias due to small sample size and allows control or experimental means to be specified as zero [2]. For several natural enemy response variables reported in primary studies (i.e., development time, mortality, time to mummification), positive values indicated lower performance on infected plants than on control plants. For these studies, d_i was multiplied by –1 to make interpretations consistent across studies. Negative values therefore indicate that natural enemies avoided or performed worse on infected plants as compared to control plants. Positive values indicate better performance on infected plants. As a rule of thumb, it is commonly accepted that $d < 0.2$, 0.5 and 0.7 correspond to small, moderate and large effect sizes, respectively.

In some papers, several experimental conditions were compared to the same control (e.g., plants infected by different pathogen species or strains compared to the same control plant). Non-independent

effect sizes may underestimate sampling variance, which was therefore corrected to account for multiple comparison to the same control using the following equation:

$$v = \frac{1}{n_{\text{treatment}}} + \frac{1}{n_{control}} + \frac{d^2}{2N}$$

where d is the Hedges' effect size, and N the total sample size of the corresponding study.

Effect sizes and their corresponding variances were calculated in R using the '*metafor*' package [3–4].

Supplementary Materials S4.

Table S1. The Number of Measured Responses Following Given Moderators; CH: consumptive effect via herbivore, NH: non-consumptive effect via herbivore and NHP: non-consumptive effect via herbivore and plant.

Pathogen Type	Type of Response	Mechanism
Bacteria (n = 17)	Performance (n = 0)	CH (n = 0)
		NH (n = 0)
		NP (n = 0)
		NHP (n = 0)
	Preference (n = 17)	NH (n = 0)
		N (n = 8)
		NHP (n = 9)
Fungus (n = 59)	Performance (n = 25)	CH (n = 14)
		NH (n = 1)
		NP (n = 9)
		NHP (n = 1)
	Preference (n = 34)	NH (n = 0)
		NP (n = 6)
		NPH (n = 28)
Virus (n = 140)	Performance (n = 115)	CH (n = 102)
		NH (n = 8)
		NP (n = 0)
		NHP (n = 5)
	Preference (n = 25)	NH (n = 13)
		NP (n = 1)
		NHP (n = 11)

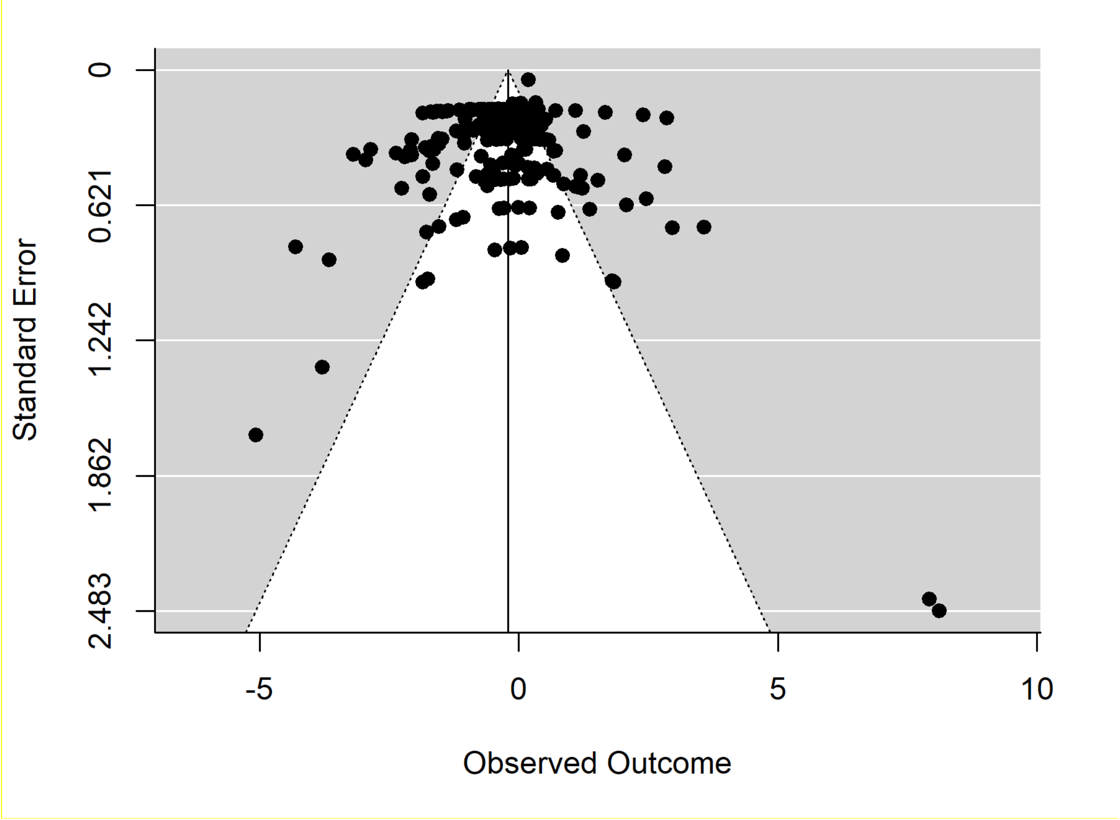

Figure S2. Funnel Plot showing the relationship between individual effect size and the standard error.

Supplementary Materials S6.

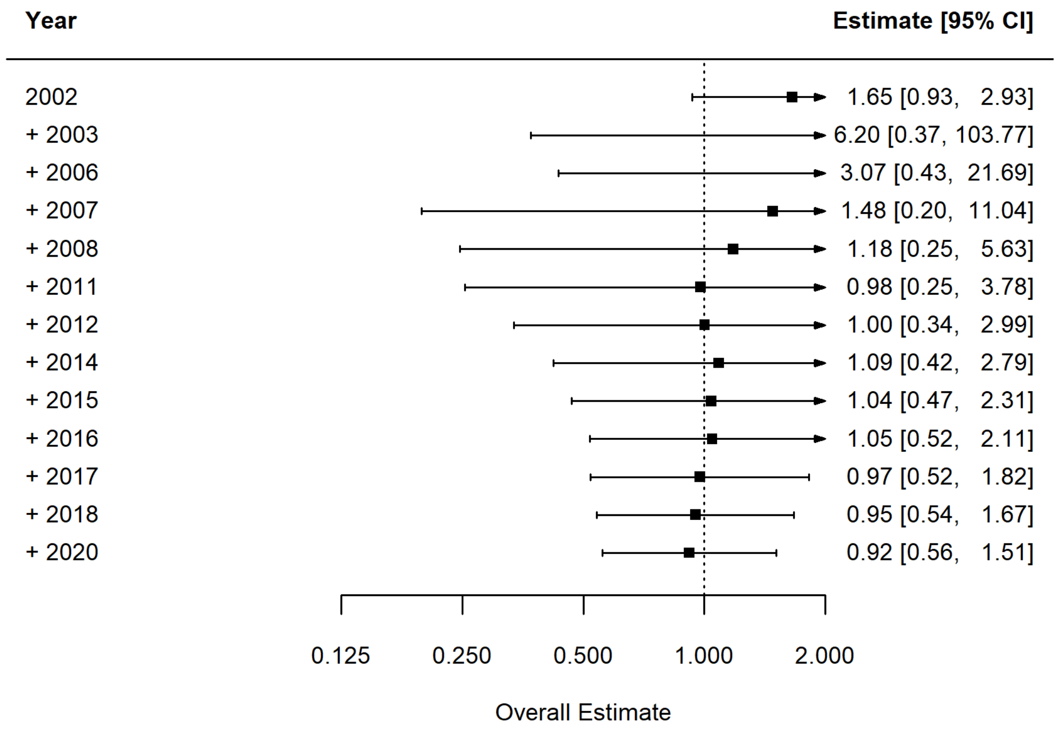

Figure S3. Temporal Trend in Combined Effect Size through Cumulative Meta-Analysis

References

1. Hedges, L.V. Distribution theory for Glass's estimator of effect size and related estimators. *J Educ. Behav. Stat.* **1981**, *6*, 107–128.
2. Koricheva, J.; Gurevitch, J.; Mengersen, K. *Handbook of meta-analysis in ecology and evolution*. Princeton University Press: Princeton, **2013**.
3. R Core Team. R: A language and environment for statistical computing. R Foundation for Statistical Computing, Vienna, **2020**.
4. Viechtbauer, W. Conducting meta-analyses in R with the metafor package. *J. Stat. Softw.* **2010**, *36*, 1–48.
